# Metagenomic Sequencing of Two Salton Sea Microbiomes

**DOI:** 10.1128/genomeA.01208-13

**Published:** 2014-01-23

**Authors:** Erik R. Hawley, Wendy Schackwitz, Matthias Hess

**Affiliations:** a Washington State University Tri-Cities, Richland, Washington, USA; b DOE Joint Genome Institute, Genome Analysis Group, Walnut Creek, California, USA; c DOE Joint Genome Institute, Microbial Genomics Group, Walnut Creek, California, USA; d Pacific Northwest National Laboratory, Chemical & Biological Process Development Group, Richland, Washington, USA; e Washington State University, Pullman, Washington, USA

## Abstract

The Salton Sea is the largest inland body of water in California, with salinities ranging from brackish freshwater to hypersaline. The lake experiences high nutrient input, and its surface water is exposed to temperatures up to 40°C. Here, we report the community profiles associated with surface water from the Salton Sea.

## GENOME ANNOUNCEMENT

Hypersaline environments are found around the globe (1) and are often populated by dense communities of halophilic prokaryotes (2). The Salton Sea, a eutrophic lake with a salinity of ~48 g liter^-1^, is the largest lake in California (3). The high nutrient loading of its influx waters facilitates algal blooms throughout the year (4), ultimately expediting the formation of anaerobic conditions associated with the microbially mediated formation of chemical species that have a negative impact on the food web (3, 5). To enhance our understanding of how environmental parameters affect the flora and fauna of the Salton Sea and its surrounding region, a thorough knowledge base of the Salton Sea microbiology is essential. Here, we report the community profile of two microbiomes associated with surface water from the Salton Sea.

Water samples were collected on 29 September and 1 October 2009 at site 1 (33°30.253′N 115°54.982′W) and site 2 (33°10.522′N 115°38.274′W), respectively. The water temperatures and pH levels of the samples were 29.9°C and 23.6°C and pH 8.09 and pH 8.20 from sites 1 and site 2, respectively. DNA was extracted using the FastDNA SPIN kit for soil from MP Biomedicals, according to the manufacturer’s protocol. The V6 to V8 region of the bacterial 16S rRNA gene was amplified using the primer set 926f/1392r and sequenced using the Roche 454 FLX+ platform (Research and Testing Laboratory, Lubbock, TX). The raw pyrosequence reads were quality filtered and analyzed using QIIME version 1.7.0 (6). Prior to filtering, the sequencing primers and barcodes were removed, allowing 1.5 mismatches to the barcode and 2 mismatches to the primer. The sequences were removed from analysis if they contained homopolymers >6 bp, were <200 bp in length, contained a quality score <25, or if they were found to be chimeric. The sequences were clustered into operational taxonomic units (OTUs) at the 97% sequence identity level using UCLUST (7), and the most abundant sequence of each OTU was chosen as a representative. The OTU representative sequences were aligned using PyNAST (8) and then filtered to remove common gaps. The reference sequences of each OTU were taxonomically classified using the RDP Classifier (9) with an 80% confidence rating against the Greengenes database (10).

A total of 1,484 and 3,341 high-quality sequences, representing 492 and 1,043 distinct OTUs, were obtained from sites 1 and 2, respectively. A total of 46 distinct phyla were identified in this study, with 34 of the phyla shared between the two samples. The two most abundant phyla detected were *Proteobacteria* (50.0% at site 1 and 52.1% at site 2) and *Bacteroidetes* (11.19% at site 1 and 8.50% at site 2). *Spirochaetes* (5.9%), *Planctomycetes* (5.1%), and unclassified bacteria (4.3%) were the next-most-abundant phyla for site 1, while unclassified bacteria (7.5%), *Planctomycetes* (4.2%), and *Cyanobacteria* (4.2%) were the next-most-abundant phyla for site 2.

Collectively, our data reveal a phylogenetic diversity and variance within the microbial communities of geospatially distinct sites from California’s largest lake, the Salton Sea.

### Nucleotide sequence accession number.

The DNA sequences from this metagenomic project have been deposited in the NCBI Short Read Archive under the accession no. SRP033722.
